# Proteomic Analysis of Three Independent Series of Sequential Cystic Fibrosis Strains in an International *Pseudomonas aeruginosa* Reference Panel Indicates Positive Selection in Late Infection Strains

**DOI:** 10.1002/mbo3.70417

**Published:** 2026-09-16

**Authors:** Joanna Drabinska, Lucia O'Connor, Caoilin McClean, Siobhán McClean

**Affiliations:** ^1^ School of Biomolecular and Biomedical Science and UCD Conway Institute of Biomolecular & Biomedical Research, University College Dublin Dublin Ireland; ^2^ School of Mathematics and Statistics, University College Dublin Dublin Ireland

**Keywords:** adaptation, cystic fibrosis, proteomics, *Pseudomonas aeruginosa*, stress responses

## Abstract

*Pseudomonas aeruginosa* is a highly diverse, adaptable Gram‐negative bacterium that thrives in many environments and is a frequent cause of chronic opportunistic infections in people with cystic fibrosis (CF). *P. aeruginosa* adapts over time of colonization to facilitate chronic infection, including loss of virulence factors; however, proteomic analyses of the adaptation to chronic infection have been limited. We previously assembled and characterized an international panel of *P. aeruginosa* strains from diverse clinical and geographical sources, including three sets of sequential CF isolates, enabling identification of conserved adaptations linked to chronic CF lung colonization. We compared the proteomes of eight strains (three early and five late infection) to assess whether common proteomic changes emerged during colonization across the sequential isolates. We identified 11 proteins showing increased abundance in late isolates in all three series, many of which are associated with virulence, regulation of virulence, or response to hypoxia. These include CF inhibitory factor repressor (CifR); WspR; 2 two‐component response regulators (PA2572 and PA3702), and transcriptional regulator (PA2551). Moreover, we identified three proteins (PA2572, PA3819, and PA5028) that showed increased abundance in all five late isolates. The probability of this being random is 5.06 × 10^−53^ and therefore, strong evidence of positive selection. The increased abundance of PA2573 and PA3819 appears to improve the fitness of *P. aeruginosa* in response to antibiotics and oxidative stress. All three proteins share a tyrosine phosphorylation motif, suggesting a common regulatory mechanism. Overall, despite substantial diversity across *P. aeruginosa*, common adaptations occur in the CF lung.

## Graphical abstract

Comparison of proteomes from three series of sequential *Pseudomonas aeruginosa* isolates from people with cystic fibrosis identified eleven proteins with increased abundance in at least one late isolate per patient. Three proteins were increased in all five late isolates, and the probability of this occurring by chance is 5.06 × 10^−53^, indicating strong positive selection. Two of the proteins appear to contribute to increased fitness to oxidative stress, and all share a tyrosine phosphorylation motif, suggesting a common regulatory mechanism.

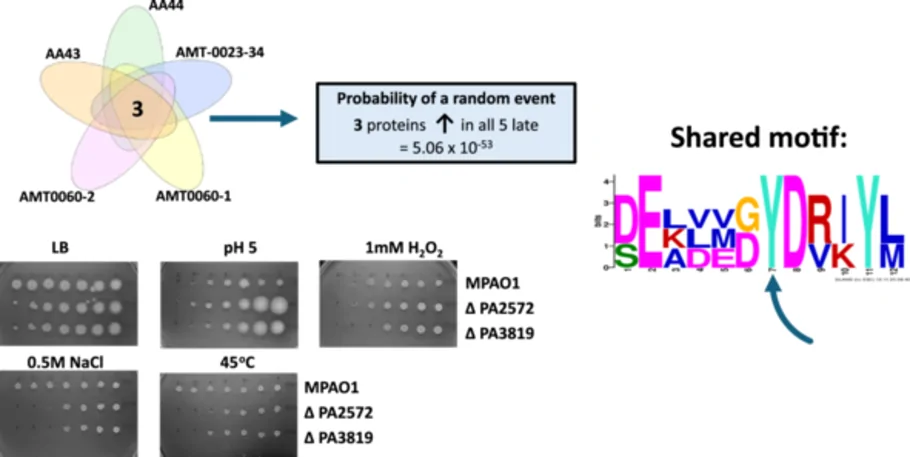

## 1 Introduction

*Pseudomonas aeruginosa* is a Gram‐negative bacterium that is a frequent cause of nosocomial and opportunistic infections, and that thrives in a wide range of environments, including man‐made water systems, contact lens solutions, and soil (Jurado‐Martin et al. 2021; Rutherford et al. 2018). The World Health Organization has listed *P. aeruginosa* as a high‐priority pathogen for which new antibiotics are currently needed (WHO 2024). It also causes chronic infections in people with cystic fibrosis (CF). Indeed, despite CF patients receiving more than 12 months of modulator therapy, *P. aeruginosa* remains problematic in a substantial cohort of patients, and persistent airway inflammation remains (Armbruster et al. 2024; Saluzzo et al. 2022). Moreover, chronic infections are not being completely cleared and are more challenging to monitor when patients are on CFTR modulators (Nichols et al. 2022; Allen et al. 2023).

It is well recognized that *P. aeruginosa* adapts over time of infection to facilitate chronic colonization (Jurado‐Martin et al. 2021; Winstanley et al. 2016; Cullen and McClean 2015; Colque et al. 2020). Common features of *P. aeruginosa* adaptation reported include loss of virulence factors, such as flagella, motility, and type III secretion system, as recently reviewed (Jurado‐Martin et al. 2021). Given the diversity of *P. aeruginosa* globally, we previously collated an international panel of 41 *P. aeruginosa* sequenced strains (De Soyza et al. 2013). We subsequently performed a detailed phenotypic analysis and comparative genomic analysis based on whole genome sequence data to define the characteristics of this international panel of strains (Freschi et al. 2018; Cullen et al. 2015). Within this panel were three independent series of sequential strains isolated from three people with CF in geographically different regions. One series of three sequential strains was acquired in Germany from an individual with CF and is designated AA2 (early), AA43, and AA44, with the latter two strains being the last isolates before the patient's death, 7.5 years later (Bragonzi et al. 2006, 2009, 2012). Another series of three strains, designated AMT 0060‐1, AMT 0060‐2, and AMT 0060‐3, were strains from a pediatric patient in Seattle (Mulcahy et al. 2010), with AMT 0060‐3 being the early strain when the patient was 7.7 years old and the other two strains being isolated 7.9 years later. Finally, a pair of strains (AMT0023‐30 and AMT0023‐34) was isolated 96 months apart from a young pediatric patient; the first strain was isolated when the patient was only 6 months old (Mulcahy et al. 2015). The 96‐month strain had 68 unique mutations relative to the first strain. In each case, the time between isolation of the early and late strains was comparable (Mulcahy et al. 2010; Tümmler 2019), at approximately 7 years (Table 1).

**Table 1:** Summary of sequential *Pseudomonas aeruginosa* isolates from three CF patients used in this study and the changes in protein abundance between early and late isolates.

Series/strain	Source	MLST (Freschi et al. 2018)	Age at first isolation (year)	Time from early strain (year)	References	Phenotype characterization (Cullen et al. 2015)						# Proteins showing changed abundance
						Virulence	Pyocyanin	Swarming	Swimming	Twitching	Alginate	
AA2	Germany	708	3.9	—	Bragonzi et al. (2006)	—	—	—	—	—	—	—
AA43				7.5		↓	↓	↓	NC	NC	NC	78
AA44				7.5		↓	↓	↓	NC	NC	NC	267
AMT0023‐30	USA	1394	0.5	—	Mulcahy et al. (2015)	—	—	—	—	—	—	—
AMT0023‐34		1394		8	Mulcahy et al. (2015)	↓	↓	↓	↓ (Abs)	↓ (Abs)	NC	182
AMT0060‐3	USA	111	7.7	—	Mulcahy et al. (2010)	—	—	—	—	—	—	—
AMT0060‐1		111		7.9		↓	↓	↓	↓	NC	NC	166
AMT0060‐2		111		7.9		↓	↓	↓ (Abs)	↓	↓ (Abs)	↑	138

Abbreviations: Abs, absent; NC, not changed.

The diversity of *P. aeruginosa* is well documented, not only between different environments but also between different CF patients and, indeed, within individual CF patients (Winstanley et al. 2016; Colque et al. 2020; Jorth et al. 2015; Darch et al. 2015; Feliziani et al. 2014). Extensive phenotype characterization performed on these sequential CF strains revealed that reduced virulence in the *Galleria mellonella* acute infection model was the only consistently altered phenotype over time of colonization in all late isolates, an adaptation described in other reports (Cullen et al. 2015; Bragonzi et al. 2009; Lorè et al. 2012), while reduced pyocyanin was observed in four out of five late strains relative to the earlier strain, with AMT0060‐1 being the exception (Table 1). Consequently, this well‐characterized strain panel presented an excellent opportunity to seek potential conserved alterations in the proteome that may be involved in driving chronic colonization in the CF lung. Thus, we compared the proteomes of all eight strains to examine whether there were any adaptations that might be common to chronic *P. aeruginosa* and highlight any conserved evolutionary mechanism(s).

## 2 Results and Discussion

### 2.1 Eleven Proteins Were Changed in Abundance in Late Strains of All CF Sequential Series, Highlighting Positive Selection During Chronic Infection

Proteomic analysis of each series identified considerable changes in protein abundance between early and late‐stage strains (Supporting Information S2: Appendix 4, Figure S2). A total of 138 proteins had significantly changed abundance by 1.5‐fold or more between the AMT0060‐2 late strain and the AMT0060‐3 early strain, while 166 proteins had changed between the AMT0060‐1 late strain and the AMT0060‐3 strain (Table 1). Of these, 57 proteins were common to both late strains, indicating a degree of shared adaptation in this series and as would be expected for strains with a shared ancestor. In the case of the AA2 series, a total of 78 proteins were altered in abundance from the AA2 strain to the AA43 strain, while 267 proteins were altered by 1.5‐fold or more between AA2 and AA44. Of these 267 proteins, there were only 30 proteins that showed changes in abundance in both late strains in this series. Although only 78 proteins showed altered abundance from AA2 and AA43, 38% of these changes were maintained in the other late strain, AA44. Finally, a total of 182 proteins were altered in abundance by 1.5‐fold or more between AMT‐0023‐30 and AMT0023‐34. Given the diversity across *P. aeruginosa*, we specifically sought proteins that were significantly changed in abundance in two or more series of strains to identify possible adaptations that might be conserved during host adaptation within the species.

**Figure 1:**
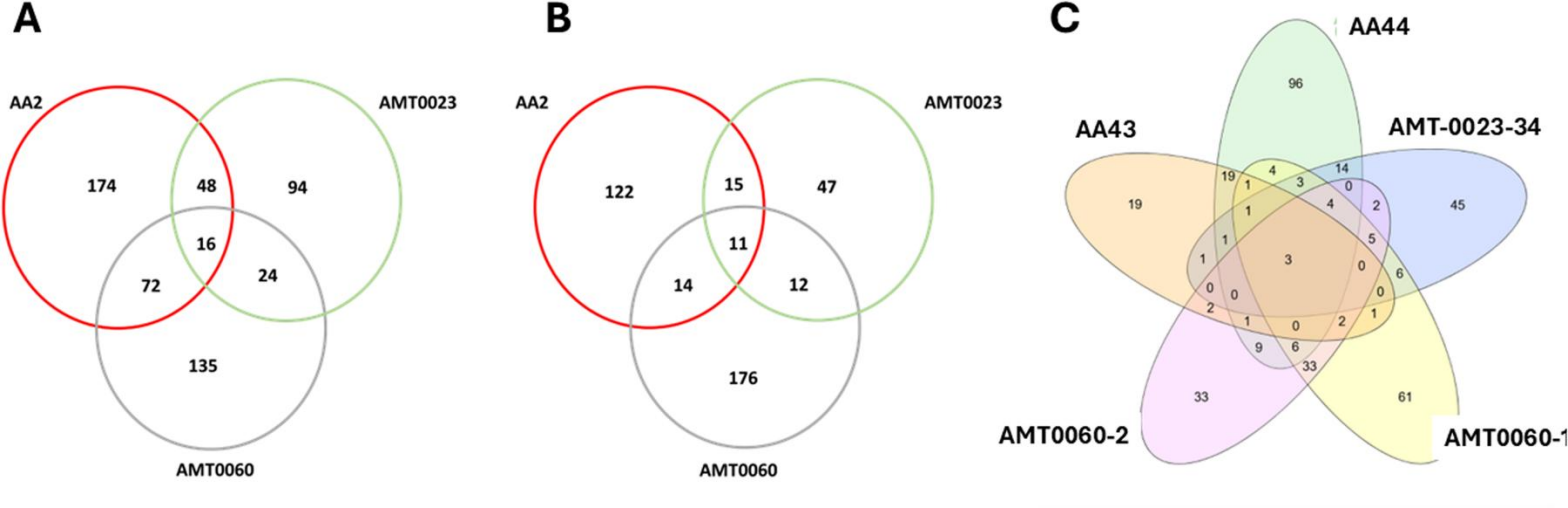
Venn diagrams indicating the changes in protein abundance in each independent series of *Pseudomonas aeruginosa* sequential strains. Numbers represent the number of proteins in the late isolate(s) relative to the respective early strain in each series. (A) All proteins changed in abundance (i.e., increased or decreased) by 1.5‐fold or more in any series of isolates (*p* < 0.05). (B) The number of proteins that showed an increase in abundance by 1.5‐fold or more between early and late isolate(s) in any given series (*p* < 0.05). (C) The number of proteins that show increased abundance in any of the five late isolates (*p* < 0.05).

Detailed analysis of the eight proteomes of the three series of strains indicated that only 16 proteins in total were found to have altered abundance across all three series (Figure 1A). Of these, 11 showed consistent changes, with all exhibiting increased abundance in the later strains of each series (Figure 1B), and consequently, we focussed our analysis on this subset (Table 2). They include proteins involved in cell adhesion (PA3702), cell division (PA1462), 2 two‐component response regulators (PA2572 and PA3702), and a transcriptional regulator (PA2551). In all cases (with the sole exception of PA2883, DUF 2897 family protein), these proteins were undetectable in the early strain, indicating that the genes were switched on over time of colonization of the lung (Table 2). Furthermore, in the case of PA2883, while it was not detected in two of three biological replicates of the AA2 early strain and was present in the third biological replicate (and its technical replicate), it was increased in abundance in AA44 relative to AA2. This protein was undetectable in the other two early strains (AMT23 and AMT0060‐3), and it is likely that this gene is switched on in the late isolates in these series also. Overall, this indicates a consistent switching on of all 11 of these genes over time of infection. Of the remaining five proteins common to all series but not showing consistent changes, both the AA2 series and AMT0023 series both showed a decrease in abundance in the later strains, while the AMT0060 series showed increased abundance of all five proteins.

**Table 2:** Proteins showing increased abundance in later strains in all three series of sequential *Pseudomonas aeruginosa* strains.

Gene ID	Protein names	GO (bioprocess/cellular location)	AA2	AA43	AA44	AMT23	AMT23	AMT0060‐3	AMT0060‐1	AMT0060‐2
PA1462	Probable plasmid partitioning protein	Cell division	**0**	**0**	**3**	**0**	**3.7**	**0**	**11**	**11**
PA2551	Probable transcriptional regulator	Regulation of DNA‐templated transcription	**0**	**0**	**11**	**0**	**15**	**0**	**3.5**	**0**
PA2572	Probable two‐component response regulator	Phosphorelay signal transduction system;	**0**	**11**	**15**	**0**	**12**	**0**	**18**	**12**
PA2679	Methyltransferase type 11 domain‐containing protein	Methyltransferase activity	**0**	**16**	**17**	**1/4**	**3.2**	**0**	**16**	**0**
PA2883	DUF2897 family protein	Membrane	**2/4**	**0**	**2.2**	**1/4**	**3.6**	**0**	**18**	**16**
PA2931	CifR	DNA binding; negative regulation of transcription	**0**	**0**	**10**	**0**	**11**	**0**	**10**	**0**
PA3084	DUF2797 domain‐containing protein	Hypothetical, unclassified	**0**	**0**	**15**	**0**	**15**	**0**	**10**	**13**
PA3271	histidine kinase (EC 2.7.13.3)	Phosphorelay signal transduction system	**0**	**0**	**16**	**0**	**13**	**0**	**3.4**	**0**
PA3702	Probable two‐component response regulator	Cell adhesion involved in single‐species biofilm formation	**0**	**0**	**12**	**0**	**16**	**0**	**15**	**14**
PA3819	Glycine zipper 2TM domain‐containing protein	Outer membrane lipoprotein protein SlyB	**0**	**12**	**19**	**0**	**12**	**1/4**	**4**	**5**
PA5028	AAA domain‐containing protein	Hypothetical; ParAB_family; Nucleoside triphosphate hydrolase	**0**	**15**	**17**	**0**	**13.6**	**0**	**17**	**13**
Not detected										
< 3										
< 8										
< 12										
< 20										

*Note:* Numbers within cells indicate Log_2_‐fold change.

The AA2 and AMT0023 series shared an additional 48 proteins that were changed in abundance in later strains, 42 of which showed consistent changes (15 were increased in abundance, while 27 were reduced in abundance). A total of 24 additional proteins were common between the two series of strains from the two US pediatric patients (AMT0023 and AMT0060) (Figure 1A). Given that there were 182 proteins showing changes in abundance in the AMT0023 series, 48% of these were also found in one of the other two sets of sequential strains, which strongly indicates that, despite the diversity inherent in *P. aeruginosa*, certain adaptation pathways are conserved over time of colonization. Moreover, a total of 72 proteins were common between the AA2 series and the AMT0060 series, with 40 of these showing consistent changes, in terms of consistently showing increased or decreased abundance in the latter strain. The other 32 proteins showed opposing changes in abundance (increased in one series of sequential strains and decreased in the other).

Overall, 144 proteins showed altered abundance in more than one series of strains (Figure 1). The consistent change in abundance of the same 11 proteins in sequential strains from three different patients suggests that these proteins have roles in the adaptation of *P. aeruginosa* to chronically colonize the CF lung. Of these 11, there were only three proteins that showed increased abundance in all five late isolates from the three series (Figure 1C and Table 2). The probability that the same three proteins would show increased abundance in all five late strains from three individual early strains is a random event, is 5.06 × 10^−53^, and consequently is very strong evidence of positive selection.

In the AA43 late isolate, 7 of the 11 proteins were not detected, and in AMT0060‐2, 4 of the 11 proteins were not detected, whereas increased abundance of these proteins was observed in the corresponding late isolates from the same series (AA44 and AMT0060‐1), which were both isolated at the same time following infection. The difference between the two late isolates in the same series is not unexpected, as microevolution within the CF lung has been well described, and divergent paths are likely (Winstanley et al. 2016; Jorth et al. 2015; Williams et al. 2015; Rossi et al. 2021). Indeed, earlier genomic characterization demonstrated substantial diversification within each of these series (Freschi et al. 2018). Although no individual protein was consistently downregulated across all late isolates, convergence was observed at the level of functional pathways. Proteins associated with virulence, including secretion systems and secreted factors, were reduced across multiple strains, consistent with the well‐described attenuation of acute virulence traits during chronic infection (Bragonzi et al. 2009; Darch et al. 2015; Rossi et al. 2021). Reductions were observed in proteins linked to lifestyle‐associated functions, including motility and surface interaction, as well as transport and envelope‐associated systems, redox and respiratory processes, and amino acid and central metabolic pathways, although the patterns of adaptation were not uniformly conserved across all strains. This is exemplified by pyocyanin production, for example. Several proteins involved in phenazine biosynthesis (e.g., PhzB, PhzF, PhzG, PhzM, and PhzS) were decreased in abundance in AA44 and AMT0023‐34, consistent with the reduced pyocyanin production previously reported (Cullen et al. 2015). However, these proteins were not decreased in abundance in the AMT0060 series. Notably, phenazine production is regulated at multiple levels beyond biosynthesis, including quorum sensing and quinolone signaling, both of which were also affected in this dataset, with decreased abundance of LasR and PQS‐associated proteins (PqsB, PqsC, PqsD, and PqsH). In addition, changes in redox‐associated proteins and transport systems may further influence phenazine production and accumulation. Together, these findings suggest that reduced pyocyanin production may arise through multiple regulatory mechanisms, rather than solely through changes in biosynthetic enzyme abundance. Overall, these findings suggest that adaptation occurs through parallel but distinct evolutionary trajectories, resulting in shared functional outcomes despite differences at the level of individual proteins.

### 2.2 Many Proteins That Showed Increased Abundance in Late Isolates Have Roles in Regulation of Virulence Factors, Response to Hypoxia, or Motility

One of the proteins that shows increased abundance in all five late strains, **PA3819,** is an outer membrane lipoprotein with a glycine zipper domain (Winsor et al. 2004). It was reported to be a putative SlyB‐like lipoprotein that is encoded in the AlgU (σ^22^) regulon and may have a role in membrane integrity (Wood and Ohman 2009). AlgU is a sigma factor that regulates the expression of alginate and is released by cell‐wall stress. PA3819 is one of eight lipoproteins activated in mucoid *P. aeruginosa*, many of which are involved in TLR signaling, in late strains (Firoved et al. 2004). Given the previous identification of alginate production in AMT0060‐2 only (Cullen et al. 2015), AlgU upregulation of PA3819 appears to be quite specific, rather than regulon‐wide.

**PA2572** is also increased in abundance in all five late strains. It is an HD‐GYP domain protein, characterized as a probable two‐component regulator which is encoded beside a chemotaxis transducer gene and is predicted to be involved in the regulation of cell motility (Winsor et al. 2004). It binds directly with the sensor, PA2573, which is co‐expressed with PA2572 under several conditions, including aerobic growth in the presence of nitrate and in bacteria co‐cultured with respiratory epithelial cells. Recent reports suggest that PA2572 and PA2573 are responsible for ExoS production and pyocyanin production (Sultan et al. 2021). It is described as an orphan chemotaxis regulator that has a role in virulence. Mutants of PA2572 or PA2573 showed considerably reduced virulence in a *G. mellonella* model, which contrasts with the observations reported for the three isolate series (Cullen et al. 2015), suggesting additional levels of regulation. PA2572 is one of three HD‐GYP proteins in *P. aeruginosa*, but unlike the other two, it does not hydrolyze c‐di‐GMP and has a negative influence on swarming. Consistent with this, swarming in BSM‐G agar was previously reported to be decreased in all five late strains (Cullen et al. 2015).

**PA5028** and **PA1462** are both cytoplasmic membrane proteins (Winsor et al. 2004) that appeared to be switched on in later strains relative to early strains in all three series, with PA5028 showing increased abundance in all five late strains. PA1462 was undetectable in AA43 but showed increased abundance in AA44 and all other late strains. It is predicted to be a cytoplasmic membrane protein that is involved in cell division (Winsor et al. 2004) and shares homology with the ATPase ParA (Kawalek et al. 2023). In *Vibrio cholerae*, ParA has been associated with chemotaxis. PA5028 is predicted to be a P‐loop containing nucleoside triphosphate hydrolase with an AAA domain (Winsor et al. 2004). It is also classified as a ParAB family protein. Interestingly, both PA5028 and PA1462 have been identified as partners for ParB (PA5562, spoOJ), a DNA‐binding protein. Par proteins form partitioning systems that contribute not only to cell division but also are global regulators of multiple proteins (Bignell and Thomas 2001; Mishra and Srinivasan 2022).

**WspR (PA3702)** is a diguanylate cyclase that stimulates c‐di‐GMP production and acts as the response regulator of the Wsp system, which showed increased abundance in all late strains except AA43. The Wsp system is a surface sensing system that responds to cell envelope stress. It negatively regulates flagellum‐dependent cell motility and promotes biofilm formation (O'Neal et al. 2022). In all three series, the protein changed from being undetectable in the early strain, again suggesting that the gene was induced in late strains in all three series. The increased abundance of WspR in all series of strains is consistent with the loss of motility widely reported for *P. aeruginosa* chronic isolates (Cullen and McClean 2015; Hogardt and Heesemann 2010; Amiel et al. 2010).

**CifR** is a TetR‐family, epoxide‐responsive repressor (CFTR inhibitory factor) which showed increased abundance in one late isolate in each of the three series. It regulates the expression of the virulence factor Cif via direct binding and repression (Ballok et al. 2012). Cif reduces apical expression of ABC transporters, including CFTR, by enhancing their polyubiquitination and lysosomal degradation. Ballok et al. have previously shown that *P. aeruginosa* CF isolates maintain CifR expression over time (Ballok et al. 2012), consistent with our observations in the three independent series of strains. Interestingly, given the diversity across *P. aeruginosa*, the fold change (FC) between the early strain and the respective late strain is equivalent, that is, 10‐ to 11‐fold in all three series.

The probable transcriptional regulator, **PA2551** is predicted to be a DNA‐binding protein which shares homology with LysR family transcriptional regulators and a component of an intricate network of transcriptional regulators. Recent reports suggest that PA2551 inhibits BrsA, a negative regulator of pyruvate metabolism and the TCA cycle. The latter has been reported to be released in response to stress and a requirement to cope with adverse conditions that can manifest in slow growth. It is possible that PA2551 acts to counteract this slowdown and maintain growth in the stressful environment of the CF lung.

**PA2679** is a hypothetical protein and predicted to have methyl transferase activity, and showed increased abundance in all late strains except AA43. Its expression was reported to be enhanced in response to hypoxia (0.4% oxygen) and moderately enhanced in response to airway epithelia (Winsor et al. 2016). Another hypothetical protein, **PA2883,** predicted to be a cytoplasmic membrane protein, was also increased in all late strains except AA43. STRING analysis suggested co‐expression with PA4608 (methyltransferase‐associated PilZ, MapZ), a c‐diGMP‐binding protein (Szklarczyk et al. 2023). The GEO expression database indicates that its expression doubled in response to hypoxia and increased dramatically in response to airway epithelia (Winsor et al. 2016). In vivo transcriptome analysis showed it is induced during infection and in response to antimicrobials. PA3084 is another hypothetical protein that was identified as a conditional essential gene for cardiomyocyte infection in the absence of amikacin (Ranjani et al. 2022). While all three hypothetical proteins were identified in all three series, neither PA2679, PA2883, nor PA3084 was identified in the AA43 strain, again highlighting a distinct evolutionary path for this specific late strain.

**PA3271** is classified as a probable two‐component sensor with kinase activity and is predicted to be a cytoplasmic membrane protein (Winsor et al. 2004). A **PA3271** transposon mutant showed upregulation of 15 genes and downregulation of 40 genes relative to the wild‐type strain (Zaoui et al. 2012). Among the downregulated genes were genes involved in virulence, that is, PchF (pyochelin synthase) and PchD (pyochelin biosynthesis protein); elastase, PvdS, FliD, flagellar capping protein, and TonB receptor, suggesting PA3271 is likely to regulate virulence. Consistent with that report, the abundance of PchF, elastase, PvdS, TonB receptor, and two flagellar proteins, FliL and FliD, was also altered in abundance in the late CF strains (Supporting Information S1: Appendix 1).

In summary, the 11 proteins that were consistently increased in abundance in the late isolates from all three series have roles in virulence or regulation of virulence factors, regulation of motility, or responses to hypoxia. While 8 of the 11 proteins were not identified in all five late strains, the probability of all these 8 proteins showing increased abundance in at least one late strain in each patient being random in a genome of approximately 5570 open reading frames (ORF) (Stover et al. 2000) is 1.6 × 10^−19^. Thus, the evolving population may have relied on the expression of these eight proteins in a model of cheating or cooperation. The consistent expression of these proteins across three independent sequential series strongly indicates that they are also critical for persistence and chronicity. It is interesting that no antibiotic resistance mechanisms are represented among the 11 proteins, despite multiple antibiotic resistance proteins showing increased abundance in all late strains. For example, MexA only showed increased abundance in the AA2 series, with dramatic increases in abundance in strains AA43 and AA44, but was not detected in the AMT 0060 series and was reduced in abundance in AMT23‐0034. The lack of consistent change in antibiotic resistance proteins may be due in part to different antibiotic regimens used to treat the patients.

**Figure 2:**
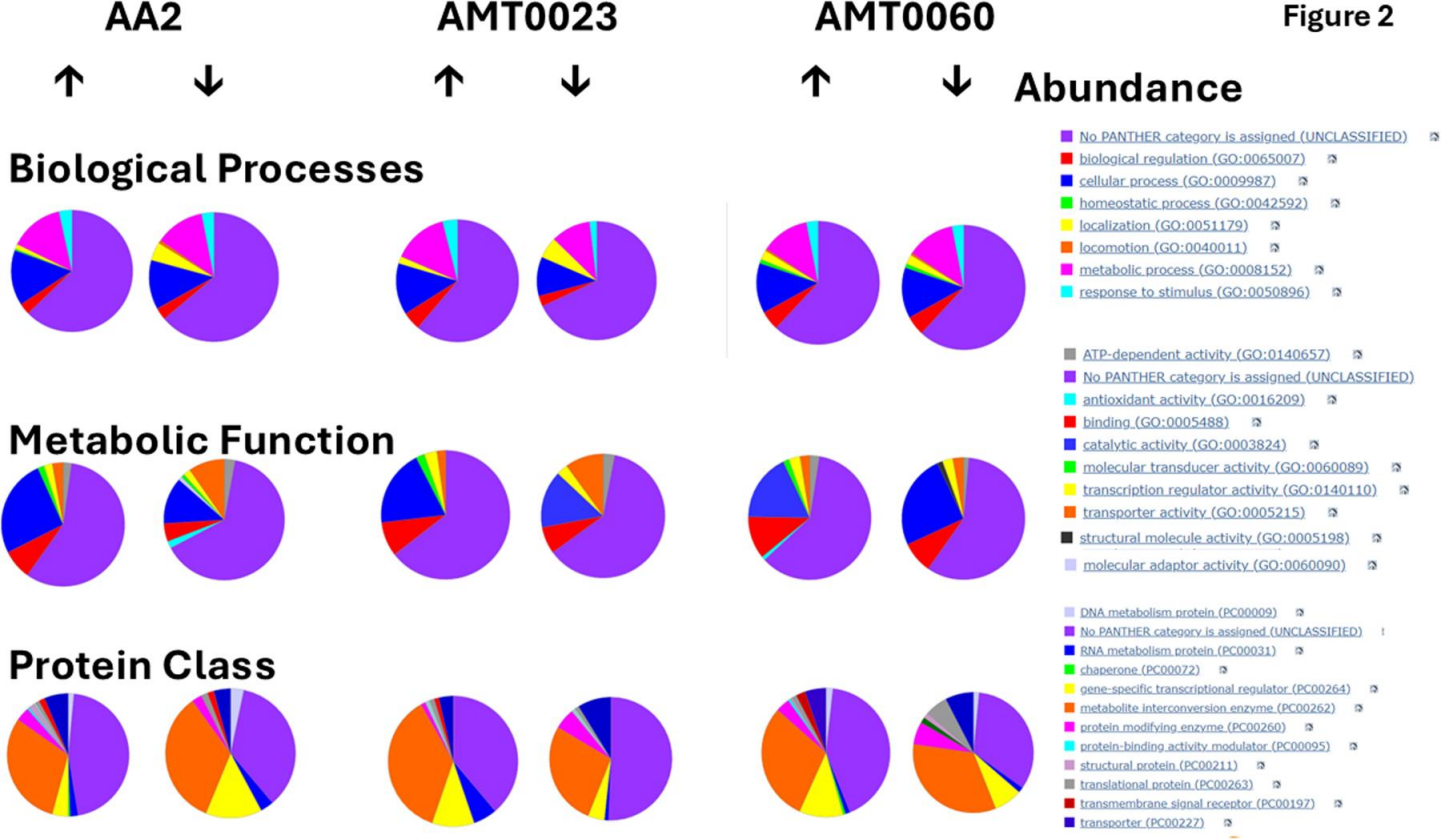
PANTHER categories of proteins that are changed in abundance in each series of sequential *Pseudomonas aeruginosa* CF strains. Up and down arrows indicate increased or decreased abundance, respectively. Biological processes, metabolic functions, and protein classes are denoted in the respective color‐coded keys.

In addition to these shared proteins, comparison of the biological processes that were associated with proteins of either increased or decreased abundance was remarkably similar across the series (Figure 2). In all cases, PANTHER could not assign a biological process to between a third and a half of the proteins; however, a comparable proportion of proteins in cellular processes, biological regulation processes, and metabolic processes were increased and decreased in all three series. There were proteins associated with homeostatic processes showing increased abundance in the AMT0060 and AA2 series but not in the AMT0023 series. Similarly, no clear metabolic function appeared to consistently show increased or decreased abundance in all three series (Figure 2B). Proteins associated with transporter activity seemed to be more represented among proteins showing reduced abundance in AMT0023 and AA2 late strains, but this was not apparent in the AMT0060 series. Comparison of protein classes did not show any consistent pattern of increased or decreased abundance across the three series. This again suggests that the specific 11 proteins that show increased abundance in all three series have an important role in the adaptation to chronic infection. Interestingly, previous studies comparing sequential isolates have not identified these 11 proteins as being consistently increased in abundance. This may be due in part to the use of less sensitive comparative proteomic methods such as 2D electrophoresis in the comparison of sequential CF isolates (Hoboth et al. 2009; Römling et al. 2013). Indeed, the proteomic analysis of sequential *P. aeruginosa* isolates has been quite limited to date.

### 2.3 PA2572 and PA3819 May Confer Increased Fitness in Response to Stress and Reduced Antibiotic Susceptibility

**Figure 3:**
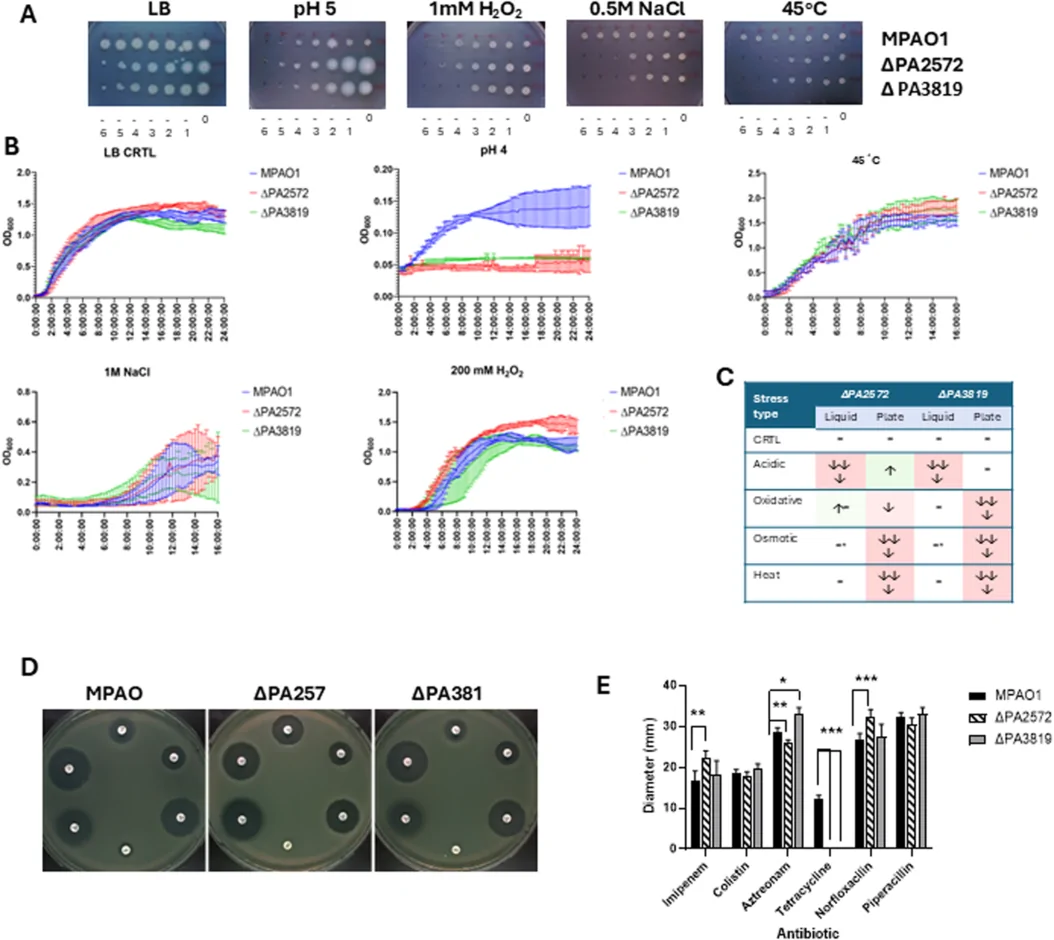
Comparison of ΔPA2572 and ΔPA3819 mutant strains with the wild type strain MPAO1 in response to stress or antibiotics. (A–C) Survival of ΔPA2572 and ΔPA3819 mutant strains in response to acidic, osmotic, temperature, or oxidative stress. (A) Survival under stress by spot dilution assay. Serial dilutions of bacterial cultures were plated, with an initial CFU of 10^6^, such that a single colony is expected in the 10^−6^ dilution. (B) Effect of environmental stresses on bacterial growth in liquid culture. Overnight cultures were diluted to a starting OD_600_ ~0.1 and grown with agitation. The experiment was repeated at least two times. (C) Summary of response to stress in plate‐ and broth‐based assays. =: no difference; ↑=: minimal increase; ↑: small increase; ↓: small decrease; ↓↓↓: large decrease. *Difference not possible to determine due to high flocculation of liquid cultures. (D, E) Antibiotic susceptibility of ΔPA2572 and ΔPA3819 mutant strains relative to MPAO1 control. (D) Representative images of plates showing changes in zones of clearance in response to a range of antibiotics, clockwise from top: imipenem, colistin, aztreonam, tetracycline, norfloxacin, piperacillin. (E) Diameters of zones of clearance in MPAO1, ΔPA2572, and ΔPA3819 mutant strains in response to antibiotics (*p ≤ 0.05, **p ≤ 0.01, ***p ≤ 0.001).

The increased abundance of PA2572, PA3819, and PA5028 in all five late strains relative to the three respective earlier strains pointed to these proteins being critical to chronic infection. Deletion mutants for the *PA2572* and *PA3819* genes were available (Jacobs et al. 2003), and neither gene was identified as essential (Lee et al. 2015). Therefore, to probe the potential roles of these two proteins further, we examined the effect of stress responses, including those that are considered typical in the CF lung, in the ΔPA2572 or ΔPA3819 gene‐deletion mutants to investigate whether increased abundance of either protein might enhance fitness in these conditions. Survival of both transposon mutants was compared with that of the wild‐type strain MPAO1 under an array of stress conditions. In the absence of stress, survival was comparable between the control strain MPAO1 and the two mutants PA2572 and PA3819 (Table 3, Figure 3A–C), as colonies were observed in the highest dilution on the control LB plates for all three strains. All three strains also showed similar growth in liquid culture with comparable growth kinetics (including the timing and duration of the lag, exponential, and stationary phases), as well as comparable maximal OD_600_ values in the stationary phase (Figure 3B). This indicates that the changes observed under stress, including decreases in OD_600_ and shifts in growth phases, reflect stress sensitivity rather than pre‐existing differences in growth. The ΔPA3819 mutant showed impaired survival under oxidative stress conditions (1 mM H_2_O_2_), while the ΔPA2572 mutant was less affected. In liquid cultures, MPAO1 and ΔPA3819 showed similar growth patterns while ΔPA2572 reached slightly higher OD_600_ (Figure 3A,B). Both strains also showed impaired survival in osmotic stress conditions (0.5 M NaCl) and elevated temperature (45°C) relative to the control. While no changes in growth in liquid culture were observed at 45°C, the effect of osmotic stress in liquid culture was inconclusive due to extensive aggregates, most likely due to biofilm formation in all three cultures in high salt. Survival of both mutant strains was unchanged in the presence of mild acid (pH 5) relative to MPAO1 when grown on plates, while growth was impaired at pH 4 in liquid cultures (Figure 3C). Overall, the absence of the *PA3819* gene impacts fitness to survive in oxidative stress conditions, while the absence of either PA3819 or PA2572 appears to impair survival in elevated salt. This strongly suggests that the increased abundance of these proteins is likely to support the ability of *P. aeruginosa* to thrive during chronic infection. To determine whether the phenotypes associated with the proteins of interest were also reflected in the clinical isolates, these were also evaluated for their growth under the stresses of acidic pH, oxidative stress, osmotic stress, and elevated temperature. As expected, distinct growth patterns were observed among the three series of strains under these stress conditions (Supporting Information S2: Appendix 4, Figure 2), however no clear pattern emerged, most likely due to the variety of proteins that were altered in the late isolates relative to their early infection counterparts.

**Table 3:** Summary of stress test analysis highlighting the lowest dilutions (10^−*x*^) at which colonies were visible in three independent experiments.

		LB			pH 5			1 mM H_2_O_2_			0.5 M NaCl 24 h			0.5 M NaCl 48 h			45°C		
		MPAO1	PA2572	PA3819	MPAO1	PA2572	PA3819	MPAO1	PA2572	PA3819	MPAO1	PA2572	PA3819	MPAO1	PA2572	PA3819	MPAO1	PA2572	PA3819
1	1	−6	−5	−6	−5	−6	−5	−6	−5	−3	−4	−3	−3	−6	−6	−6	−6	−6	−6
	2	−6	−6	−6	−6	−5	−6	−6	−5	−6	−3	−4	−3	−6	−6	−6	−6	−5	−6
2	1	−6	−6	−6	−6	−6	−6	−6	−6	−5	na	na	na	−6	−6	−6	na	na	na
	2	−6	−6	−6	−6	−6	−6	−6	−6	−5	na	na	na	−6	−6	−6	na	na	na
3	1	−6	−6	−6	−6	−6	−5	−6	−6	−6	−6	−4	−3	−6	−6	−6	−6	−4	−4
	2	−6	−6	−5	−6	−6	−6	−6	−5	−6	−6	−4	−3	−6	−5	−6	−6	−5	−5
Mean		−6.00	−5.83	−5.83	−5.83	−5.83	−5.67	−6.00	−5.50	−5.17	−4.75	−3.75	−3.00	−6.00	−5.83	−6.00	−6.00	−5.00	−5.25

Abbreviation: na, not available.

Finally, while no antibiotic resistance‐associated proteins were consistently changed in all five late strains, we examined whether these two genes confer any fitness in the context of reduced susceptibility to antibiotics commonly used in the treatment of CF patients. Increased susceptibility to imipenem, aztreonam, and norfloxacin was apparent in the ΔPA2573 mutant, while increased susceptibility to aztreonam only was observed in the ΔPA3819 mutant (Figure 3D,E). Interestingly, both mutants were resistant to tetracycline. Collectively, this indicates that the increased abundance of these two proteins, and particularly PA2573, in the late isolates may contribute directly or indirectly to the enhanced antibiotic resistance of these late strains.

### 2.4 The Increases in Protein Abundance Are Not due to Increased Gene Transcription

**Figure 4:**
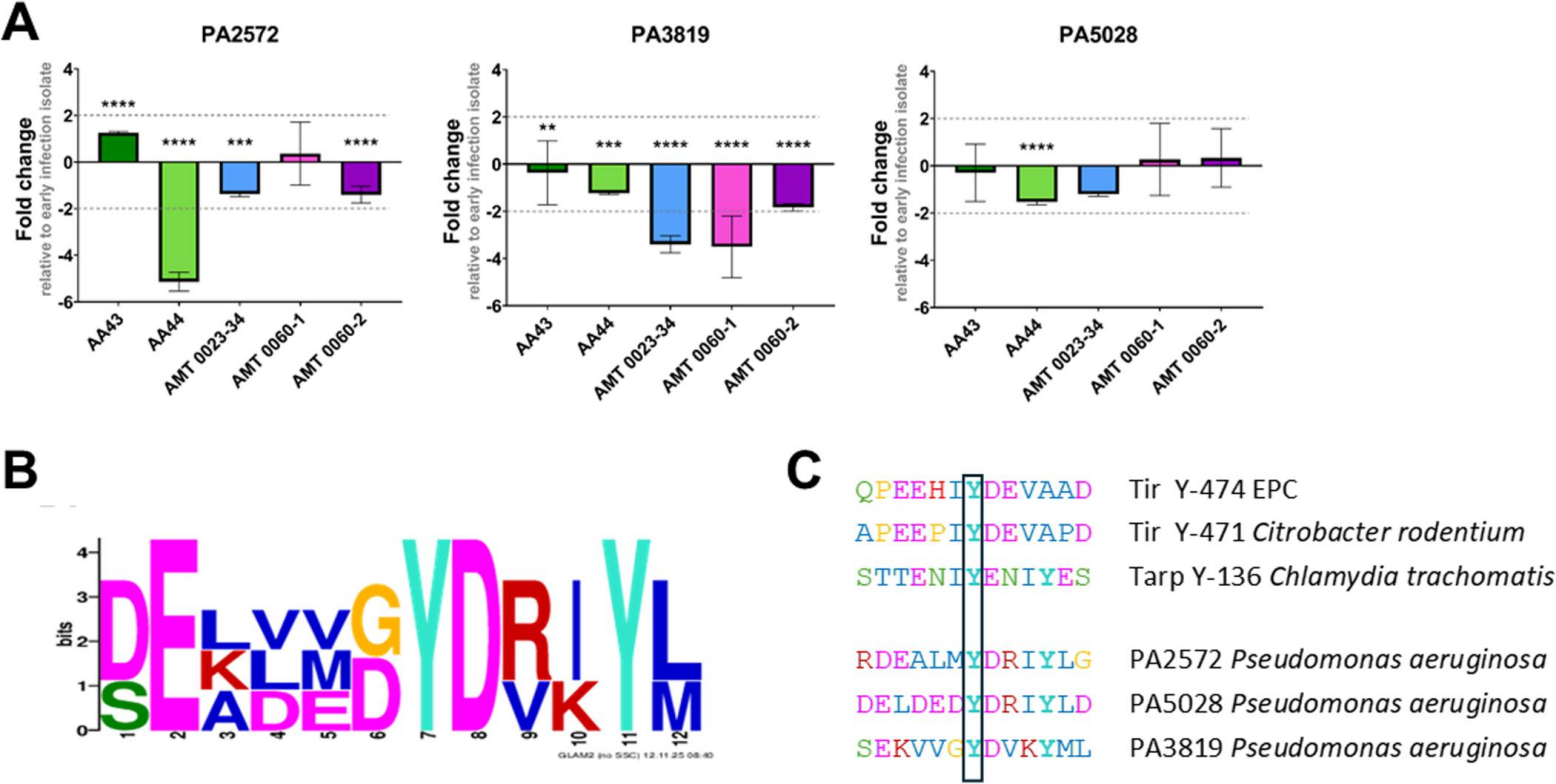
(A) Expression of *PA2572, PA3819,* and *PA5028* genes in late infection strains in comparison to the relative early infection strains. The fold‐change values were calculated using the Pfaffl equation, and the significance was calculated using unpaired *t*‐tests with the early infection strain (**p < 0.01, ***p < 0.001, ****p < 0.0001). (B) Conserved motif identified with GLAM2, as follows: red – positively charged (basic); magenta – negatively charged (acidic); green – polar (uncharged); dark blue – hydrophobic; cyan – bulky hydrophobic (aromatic and branched); yellow – structurally unique. (C) Comparison of the sequences of the identified motif in proteins of interest. The numbers on the right represent the range within the amino acid sequence.

To evaluate potential mechanism(s) for the increased abundance of the three proteins in all chronic infection isolates, we examined gene expression by qPCR. The differences in mRNA levels between early and late infection counterparts did not correspond with the observed protein changes. While transcriptional changes for all late‐infection strains were below 2‐fold for *PA5028*, expression of *PA3819* was repressed by 3.5‐fold in AMT0023‐34 and AMT0061‐1, and *PA2572* expression was repressed by 5‐fold in AA44 (Figure 4A). Although correlations between transcriptomic and proteomic expression profiles are often limited (Rogers et al. 2008; Haider and Pal 2013), the minimal transcriptional changes suggested that the increased protein abundance may be due to translational or post‐translational differences. To explore this further, we examined the amino acid sequences of PA2572, PA3819, and PA5028 for potential post‐translational modification sites known to influence protein stability (Macek et al. 2019). We identified multiple phosphorylation sites in each of the three proteins using NetPhosBac (v1.0) (Miller et al. 2009) together with glycosylation and N‐myristoylation sites ScanProsite (de Castro et al. 2006) that are predicted to be associated with protein stability (Supporting Information S1: Appendix 2). A search for conserved motifs across the three proteins identified a conserved **[DS]E[AKL][LVD][MVE][GD]YD[RV][IK]Y[LM]** motif in all three proteins of interest (Figure 4B), which shows remarkable similarity to tyrosine phosphorylation sites in other Gram‐negative species (Backert and Selbach 2005). Specifically, the tyrosine residue is located within an acidic region, with an aspartate immediately in the +1 position (C‐terminal side), an aliphatic residue in the +2 or +3 position, and other acidic residues (aspartate and/or glutamate) within the N‐terminal side (−1 to −6). This motif is shared by the tyrosine phosphorylation sites of Tir (translocated intimin receptor) in enteropathogenic *Escherichia coli* and *Citrobacter rodentium* and of Tarp (translocated actin‐recruiting phosphoprotein) in *Chlamydia trachomatis* (Figure 4C) (Backert and Selbach 2005). Tyrosine phosphorylation regulates bacterial virulence‐associated traits (Cozzone 2005; Whitmore and Lamont 2012), although tools for predicting tyrosine phosphorylation in bacteria remain limited. Nevertheless, the observed similarity suggests a shared modification site across the three proteins of interest, which may ultimately contribute to their increased abundance in response to factors associated with chronic infection.

## 3 Conclusion

Given the diversity across *P. aeruginosa* strains and isolates, not only between hosts, but also within an individual host, the increased abundance of eleven proteins in the late strains of all three independent series of sequential strains is clear evidence of positive selection of these proteins. It is likely that there are complex mechanisms at play in the adaptation to chronic infection, and undoubtedly, there may also be interplay between these proteins and pathways. Thus, this investigation of the individual proteins provides a snapshot of the overall dynamic adaptation process. Nevertheless, the increased abundance of PA2573 and PA3819 is associated with improved fitness of *P. aeruginosa* in response to antibiotics and oxidative stress, respectively, at least in part. Additional studies on these two proteins, including analysis of targeted deletion mutants and respective complement strains, would further validate their roles. Indeed, each of the 11 proteins identified represents a target for future adjuvant therapy to impair the development of chronic infection. Consequently, a detailed evaluation of the roles of these proteins in the adaptation process is warranted.

## 4 Materials and Methods

### 4.1 Bacterial Strains

The *P. aeruginosa* AA2, PAAA43, AA44, AMT0023‐30, AMT0023‐34, AMT0060‐1, AMT0060‐2, and AMT0060‐3 strains are well‐characterized strains from the International *P. aeruginosa* panel (De Soyza et al. 2013) (Table 1). The *P. aeruginosa* MPO1 and the respective *ΔPA2572* and *ΔPA3819* mutant strains were obtained from the sequence‐verified two‐allele transposon mutant library (Held et al. 2012), available from the Salipante lab, University of Washington. All strains were routinely grown in LB broth (Sigma‐Aldrich, St. Louis, MO, USA) at 37°C with orbital agitation (200 rpm) or plated on LB agar (Sigma‐Aldrich, St. Louis, MO, USA) and cultured at 37°C.

### 4.2 Whole Cell Proteome Analysis

Whole cell lysates were extracted from cultures by resuspending in 1 mL of ice‐cold lysis buffer containing 40 mM Tris‐HCl (pH 7.8) and 1× cOmplete, EDTA‐free protease inhibitor cocktail (Roche, Basel, Switzerland). Cells were disrupted by a sonication with a sonic dismembrator and microtip 6 mm probe (model 505; Fisherbrand, Thermo Fisher Scientific, Waltham, MA, USA) (set with 20% output power using eight 10 s bursts on ice). Cell debris and any remaining intact cells were pelleted using centrifugation (12,000*g* for 30 min). Supernatants were transferred into fresh tubes, and the proteins were precipitated with four volumes of methanol and incubated at −80°C overnight. The samples were centrifuged at 12,000*g* for 30 min at 4°C, supernatants were removed, and the protein pellets were resuspended in 200 μL of resuspension buffer containing 8 M urea, 50 mM Tris‐HCL (pH 8.0), before 1 M 1,4‐dithiothreitol was added to each supernatant (10 μL/mL lysate) and incubated at 56°C for 30 min, followed by the addition of 1 M iodoacetamide (55 μL/mL lysate) which was incubated in darkness at room temperature for 20 min. Lysates were dialyzed in SnakeSkin tubing with a cut‐off of 3.5 kDa (Thermo Fisher Scientific, Waltham, MA, USA) against 100 mM ammonium bicarbonate overnight, with stirring at 4°C, followed by at least four more hours of incubation with fresh volumes of ammonium bicarbonate. Trypsin (400 ng/μL) (Sigma‐Aldrich, St. Louis, MO, USA) was added to the dialyzed protein (5 μL/100 μL protein), and tubes were incubated overnight at 37°C. Aliquots from each sample were placed in fresh tubes, and samples were dried in a vacufuge concentrator system (model 5301; Eppendorf, Hamburg, Germany) at 65°C and resuspended in 20 μL of ZipTips resuspension buffer containing 0.5% trifluoroacetic acid (TFA) in MilliQ water. ZipTips (Merck Millipore, Burlington, MA, USA) were used for peptide purification as per the manufacturer's instructions. Nanodrop spectrophotometer DS‐11+ (DeNovix, Wilmington, DE, USA) was used for peptide concentration measurements. The samples were analyzed on a Bruker TimsTOF Pro mass spectrometer connected to an Evosep One chromatography system. Peptides were separated on an 8 cm analytical C18 column (Evosep, 3 µm beads, 100 µm ID) using the pre‐set 33 samples per day gradient on the Evosep one. A trapped ion mobility (TIMS) analyzer was synchronized with a quadrupole mass filter to enable the highly efficient PASEF (Parallel Accumulation Serial Fragmentation acquisition) procedure with acquisition rates of 100 Hz. The accumulation and ramp times for the TIMS were both set to 100 ms, with an ion mobility (1/k0) range from 0.62 to 1.46 Vs/cm. Spectra were recorded in the mass range from 100 to 1700 m/z. The precursor (MS) Intensity Threshold was set to 2500, and the precursor Target Intensity was set to 20,000. Each PASEF cycle consisted of one MS ramp for precursor detection followed by 10 PASEF MS/MS ramps, with a total cycle time of 1.16 s.

Protein identification and label‐free quantitative (LFQ) analysis were conducted using MaxQuant (v 2.4.2.0, https://maxquant.org/) by searching against the reference strain *P. aeruginosa* PAO1 (ATCC 15692). For protein identification, the following search parameters were used: trypsin was the digesting enzyme with up to two missed cleavages allowed for; oxidation on methionine and acetylation on the N terminus were selected as variable modifications with a fixed modification of carbamidylation on cysteine; Label Free Quantitation (LFQ) and Match Between Runs options were selected. FDR for proteins and peptides was set at 1%.

Three biological replicates per strain were analyzed, with one technical replicate included for a randomly selected sample. Missing LFQ intensities were imputed with a fixed value of 1. Mean intensities were calculated separately for each strain, followed by FC calculation. Pairwise comparisons were performed using a two‐tailed unpaired Student's *t*‐test. Proteins with *p*‐values ≤ 0.05 were considered significantly altered in abundance.

### 4.3 Stress Sensitivity Survival Assays

Overnight cultures of *P. aeruginosa* strains were diluted to a CFU/mL of 10^8^, and a range of serial dilutions was prepared. Suspensions (1 μL) were plated on one of the following: LB agar control plates; LB agar supplemented with 0.5 M NaCl (osmotic stress); LB agar supplemented with 1 mM H_2_O_2_ (oxidative stress); or LB agar, pH 5 (acidic stress). The plates were incubated at 37°C for 24 h. Temperature stress was determined by incubation of an LB agar plate at 45°C for 24 h. Changes in sensitivity were determined by determining the lowest dilution at which bacterial growth was observed. The experiment was repeated independently three times.

### 4.4 Effect of Environmental Stresses on Bacterial Growth in Liquid Culture

Overnight cultures of *P. aeruginosa* strains (sequential isolates, MPA01 or mutant strains) were diluted to an OD600 ~0.1 in LB or supplemented media, and 200 µL of culture was pipetted into each well of a 96‐well plate, with at least two technical replicates per condition. LB media was used as the control and to assess heat stress; LB media supplemented with 1 M NaCl was used to assess osmotic stress; LB media supplemented with 200 mM H_2_O_2_ was used to assess oxidative stress; and LB media adjusted to pH 4 was used to assess acidic stress. For temperature stress, the 96‐well plate was incubated at 45°C, whereas all other conditions were incubated at 37°C with orbital agitation in a shaker incubator. Bacterial growth was assessed. The experiment was repeated at least two times independently.

### 4.5 Determination of Antibiotic Resistance

Overnight bacterial cultures were diluted in 10 mL of fresh LB to an OD600 equal to 0.1. Bacteria were spread evenly on Mueller–Hinton agar plates (Neogen, Lansing, MI, USA) using cotton swabs, in three directions, to give a homogenous lawn. Antibiotic disks (Oxoid, Hampshire, UK) were placed on the surface of the agar, and the plates were incubated overnight in normoxic conditions at 37°C. Growth inhibition zone diameters were measured at least two points, and the mean was used for statistical analysis. Each experiment was repeated three times.

### 4.6 Real‐Time PCR

RNA was extracted from 1 mL of overnight bacterial cultures that had been immediately added to two volumes of Bacteria Protect Reagent (Qiagen, Hilden, Germany) and pelleted. Cell pellets were lysed in TE buffer (10 mM Tris·Cl, 1 mM EDTA, pH 8.0) containing 15 mg/mL lysozyme and 20 μL Proteinase K (Thermo Fisher Scientific), and total RNA was isolated using the RNeasy Mini Kit (Qiagen) according to the manufacturer's protocol, followed by an additional purification step using RNeasy MinElute Cleanup Kit (Qiagen). RNA concentration and purity were assessed using a spectrophotometer (Denovix). Genomic DNA was removed by treating the RNA with the RapidOut DNA Removal Kit (Thermo Fisher Scientific). Subsequently, 1 µg of DNA‐free RNA was used for cDNA synthesis with QuantiTect Reverse Transcription Kit (Qiagen). Reverse transcription‐negative (−RT) controls were prepared to confirm the absence of genomic DNA contamination.

Quantitative PCR was performed with gene‐specific primers (Supporting Information S1: Appendix 3) and a PowerUP SYBR Green Master Mix (Applied Biosystems, xx) on a QuantStudio 7 Flex Real‐Time PCR System (Applied Biosystems). Primer efficiencies for both target and reference housekeeping (*nadB*) genes were determined from a standard curve of serial cDNA dilutions and were incorporated into the analysis. Reactions were performed using a 1:5 dilution of cDNA template according to the manufacturer's instructions. Relative gene expression was calculated using the Pfaffl method using the *nadB* housekeeping gene, and statistical significance of differences between early and late infection strains was determined using Δ*C*_t_ values (Pfaffl 2001).

### 4.7 Statistical and Probability Analysis

Statistical analysis was performed by one‐way analysis of variance using Prism software with the Kruskal–Wallis test when the data did not pass the Shapiro–Wilk normality test. All graphs were prepared using GraphPad Prism 8.0.2.

To determine the probability of the three proteins showing increased abundance in all five late isolates undergoing the same adaptation, the following calculation was performed:

$${\left(\mathrm{reciprocal}\;\mathrm{of}\;(\#\;\mathrm{ORFs}\;\mathrm{choose}\;3\;\mathrm{proteins})\right)}^{\mathrm{number}\;\mathrm{of}\;\mathrm{isolates}},$$

where the number of ORFs is 5570 in *P. aeruginosa* PaO1 (Stover et al. 2000). The probability of eight proteins showing increased abundance in at least one late isolate from each patient was calculated as:

$$[1-(\#(\mathrm{ORFS}-1)\;\mathrm{choose}\;8\;\mathrm{proteins})/{{\left(\#\mathrm{ORFS}\;\mathrm{choose}\;8\;\mathrm{proteins}\right)}^{\mathrm{number}\;\mathrm{of}\;\mathrm{late}\;\mathrm{isolates}}]}^{\;8\;\text{proteins}}.$$

## Author Contributions

**Joanna Drabinska:** conceptualization, investigation, methodology, formal analysis, writing – review and editing. **Lucia O'Connor:** methodology, formal analysis, writing – review and editing. **Caoilin McClean:** methodology, formal analysis, writing – review and editing. **Siobhán McClean:** conceptualization, investigation, formal analysis, funding acquisition, writing – original draft, writing – review and editing.

## Ethics Statement

The authors have nothing to report.

## Conflicts of Interest

The authors declare no conflicts of interest.

## Supporting information


Supporting File 1

Supporting File 2

## Data Availability

The mass spectrometry proteomics data have been deposited with the ProteomeXchange Consortium via the PRIDE (Perez‐Riverol et al. 2019) partner repository under the dataset identifier PXD073355.
