# Supplementary material for: Proteomic Analysis of Three Independent Series of Sequential Cystic Fibrosis Strains in an International Pseudomonas aeruginosa Reference Panel Indicates Positive Selection in Late Infection Strains
Source: Microbiologyopen. 2026 Sep 16;15(5):e70417. doi: 10.1002/mbo3.70417 (PMC13583184; doi:10.1002/mbo3.70417)
Supplement: Supplementary file 1 [file MBO3-15-e70417-s001.docx]

**Appendix 4.**

**Supplementary Figure 1.** Volcano plots representing the comparison of protein abundance in late infection isolates in relevance to the corresponding early infection isolate. The volcano plots have been generated using VolcaNoseR (huygens.science.uva.nl/VolcaNoseR/)[1]. The proteins increased in abundance (p-value ≤0.05, fold change ≥ 1.5) are represented in red and the proteins decreased in abundance are presented in blue (p-value ≤0.05, fold change ≤ -1.5) .

**Supplementary Figure 2. Comparison of late infection strains with relative early infection strains in response to stress**. **A)** Growth patterns of early and late clinical isolates in response to acidic, osmotic, temperature or oxidative stress. The overnight cultures were diluted to OD_600_ ~0.1 and grown with agitation. **B)** Summary of resistance of late infections strains in comparison to the relative early infection strain in liquid culture.

1.	Goedhart, J. and M.S. Luijsterburg, *VolcaNoseR is a web app for creating, exploring, labeling and sharing volcano plots.* Sci Rep, 2020. **10**(1): p. 20560.
